# Flow estimation solely from image data through persistent homology analysis

**DOI:** 10.1038/s41598-021-97222-6

**Published:** 2021-09-09

**Authors:** Anna Suzuki, Miyuki Miyazawa, James M. Minto, Takeshi Tsuji, Ippei Obayashi, Yasuaki Hiraoka, Takatoshi Ito

**Affiliations:** 1 Institute of Fluid Science, Tohoku University, Sendai, 980-8577 Japan; 2 Department of Civil and Environmental Engineering, University of Strathclyde, Glasgow, UK; 3 Department of Earth Resources Engineering, Kyushu University, Fukuoka, 819-0385 Japan; 4 International Institute for Carbon Neutral Energy Research (WPI-I2CNER), Kyushu University, Fukuoka, 819-0385 Japan; 5 Cyber-Physical Engineering Information Research Core (Cypher), Okayama University, 3-1-1 Tsushima-naka, Kita-ku, Okayama, 700-8530 Japan; 6 Kyoto University Institute for Advanced Study, ASHBi, Kyoto University, Kyoto, 606-8501 Japan

**Keywords:** Hydrogeology, Applied mathematics

## Abstract

Topological data analysis is an emerging concept of data analysis for characterizing shapes. A state-of-the-art tool in topological data analysis is persistent homology, which is expected to summarize quantified topological and geometric features. Although persistent homology is useful for revealing the topological and geometric information, it is difficult to interpret the parameters of persistent homology themselves and difficult to directly relate the parameters to physical properties. In this study, we focus on connectivity and apertures of flow channels detected from persistent homology analysis. We propose a method to estimate permeability in fracture networks from parameters of persistent homology. Synthetic 3D fracture network patterns and their direct flow simulations are used for the validation. The results suggest that the persistent homology can estimate fluid flow in fracture network based on the image data. This method can easily derive the flow phenomena based on the information of the structure.

## Introduction

Fluid flow processes are ubiquitous in the world, and most are governed by the geometry and nature of the surrounding structures. In particular, recent miniaturization of artificial devices has led to the need for understanding and controlling flow in finer structures. It is also attracting attention to understand flow behaviors in complex fracture networks in developments of natural resources, as in the case of shale gas and geothermal developments.

It has been a long-term scientific challenge to predict flow behavior of porous media from structural properties. Permeability is a key parameter for examining flow phenomena in porous media^1^. Permeability cannot be determined only from structure data, and needs to be obtained from laboratory experiments or numerical fluid flow simulations. In contrast, porosity is a parameter that is often used to characterize the structures. The porosity–permeability correlation has been studied extensively in the literature to estimate permeability using porosity (so-called Kozeny–Carman equation)^2,3^. This Kozeny–Carman equation provides a relationship between structure and flow. However, no matter how many voids there are, if they are not connected, water cannot flow. Therefore, the Kozeny–Carman equation does not always work. The correlation has been modified to represent real phenomena by adding parameters such as fractal dimension, and tortuosity^2^. These additional parameters can only be determined by fitting, which is not the best way to go about flow prediction based on structural information.

Let us also consider flow in a channel from an inlet to an outlet. The Hagen–Poiseuille equation is a physical law that describe a steady laminar flow of a viscous, incompressible, and Newtonian fluid through a circular tube of constant radius, *r.* This is an exact solution for the flow, can be derived from the (Navier–) Stokes equations, and is another way of expressing the relationship between structure and flow. Using Darcy’s law, a representative permeability, *K*_HP_ [m^2^], for the capillary can be calculated depending only on the radius:

1 $$ K_{HP} = \frac{{r^{2} }}{8} $$

Similarly, for flow in a fracture bounded by two smooth, parallel walls, the permeability, *K*_CL_ [m^2^], can be calculated depending only on the aperture, *h* [m]:

2 $$ K_{CL} = \frac{{h^{2} }}{12} $$

Since the flow rate is proportional to the cube of the fracture aperture, this relationship between flow and aperture is well-known as the “cubic law” ^4–7^.

Equations (1) and (2) are only ways to obtain a simplified analytic solution to describe the relationship between the flow and structures. The approaches using Eqs. (1) and (2) are another way to predict permeability from structural properties than the Kozeny–Carman equation.^8^. In natural rocks, there is not always a single fracture, but multiple fractures that form a network. Thus, it is necessary to understand not just an individual fracture, but how channels are connected from an inlet to an outlet in whole networks. There have been many studies focusing on networks, but most of the parameters used to describe the structure are probabilistic variables that capture individual fractures, and no suitable parameters have been found yet to evaluate the flow of the entire networks^9^.

Topology, a branch of modern mathematics, is good at roughly investigating the connectivity of shapes. Topology focuses on the properties (called topological properties or topological invariants) that are preserved when some form (shape or space) is continuously deformed (stretched or bent, but not cut or pasted). Topology can extract global features that are difficult to capture with machine learning and convolutional neural networks, so it is promising as a complementary feature to extract image information that cannot be detected with other methods. It can be applied to volumetric data as well, so it can pick up information that has been missed in one-way slice-by-slice analysis common to many forms of data processing.

Several studies used topological invariants to describe pore‐scale structures in porous materials and fracture networks^10,11^. The Minkowski functionals can be interpreted as area, perimeter, or the Euler characteristic, which is a topological constant and were used to link to hydraulic properties^12,13^. Scholz et al.^14^ showed an empirical expression of permeability with the Minkowski functionals. Liu et al.^15^ showed the correlation of relative permeability to one of the topological invariants called Euler characteristic. Armstrong et al.^16^ reviewed the theoretical basis of the Minkowski functionals and its application to characterize porous media. Counting the number of holes using topological invariants like they did is a clue to the shape of the object, and the "essential information" can be extracted well. On the other hand, topology too narrowly focuses on the essential information, it also discards a lot of information, such as size of the pore space. The size information, such as radii of tube or apertures of fractures in Eqs. (1) and (2), must be detected to determine permeability derived analytically. Therefore, previous studies had to add the size information in other ways.

Homology is a standard technique for identifying a topological space. In particular, the concept of homology has traditionally played a role in feature extraction focusing on the existence of “holes”. Here, the “hole” structure can be regarded as a connected flow channels from an inlet to an outlet. It is expected that topology can be used to detect such connected flow channels.

By tracking the sequence of topological spaces, namely, by recording how long homological features persist, we can add information about the size and length of the holes. This can give us a quantitative indication of the size of the holes and the amount of space available, which is called persistent homology. Persistent homology is one of the most important tools in topological data analysis and is expected to compute geometric and topological features of various shapes with ease of computation^17–20^. Thus, this has been applied in several research fields^21–25^, and is also beginning to be used in the analysis of porous materials^26–31^.

At this point, in contrast to topological invariants, persistent homology can provide a lot of information that we might need, but it is difficult to interpret the parameters of persistent homology themselves^27–30^. Ushizima et al.^26^ estimates permeability of porous rocks by using Reeb graphs to ﻿represent the pore networks. They use persistent homology to distinguish between significant and “noisy” pore spaces, and to supplement the Reeb graphs. Their paper did not go into quantitative evaluation, but focused on qualitative evaluation and visualization. As mentioned before, the “hole” structure that is characterized by topology, can be regarded as connected flow channels from an inlet to an outlet. The aim of our study is to detect the flow channels by persistent homology. Suzuki et al.^31^ proposes a method to detect flow channels in 2D images from persistent homology through image processing. By using their image processing procedure, persistent homology is expected to detect such connected flow channels in complex fracture networks and would also provide their size information such as apertures to predict the permeability.

In this study, we applied persistent homology to estimate permeability in fracture networks based on image data. Persistent homology was used to detect the number of flow channels and their apertures in the networks. Synthetic fracture networks were generated, and direct flow simulation was conducted. Permeability derived from persistent homology and simulation results were compared. We applied the proposed method to several published image data and discussed the applicability of permeability estimation based on persistent homology.

## Results

### Detection of flow channels from persistent homology analysis

**Figure 1:**
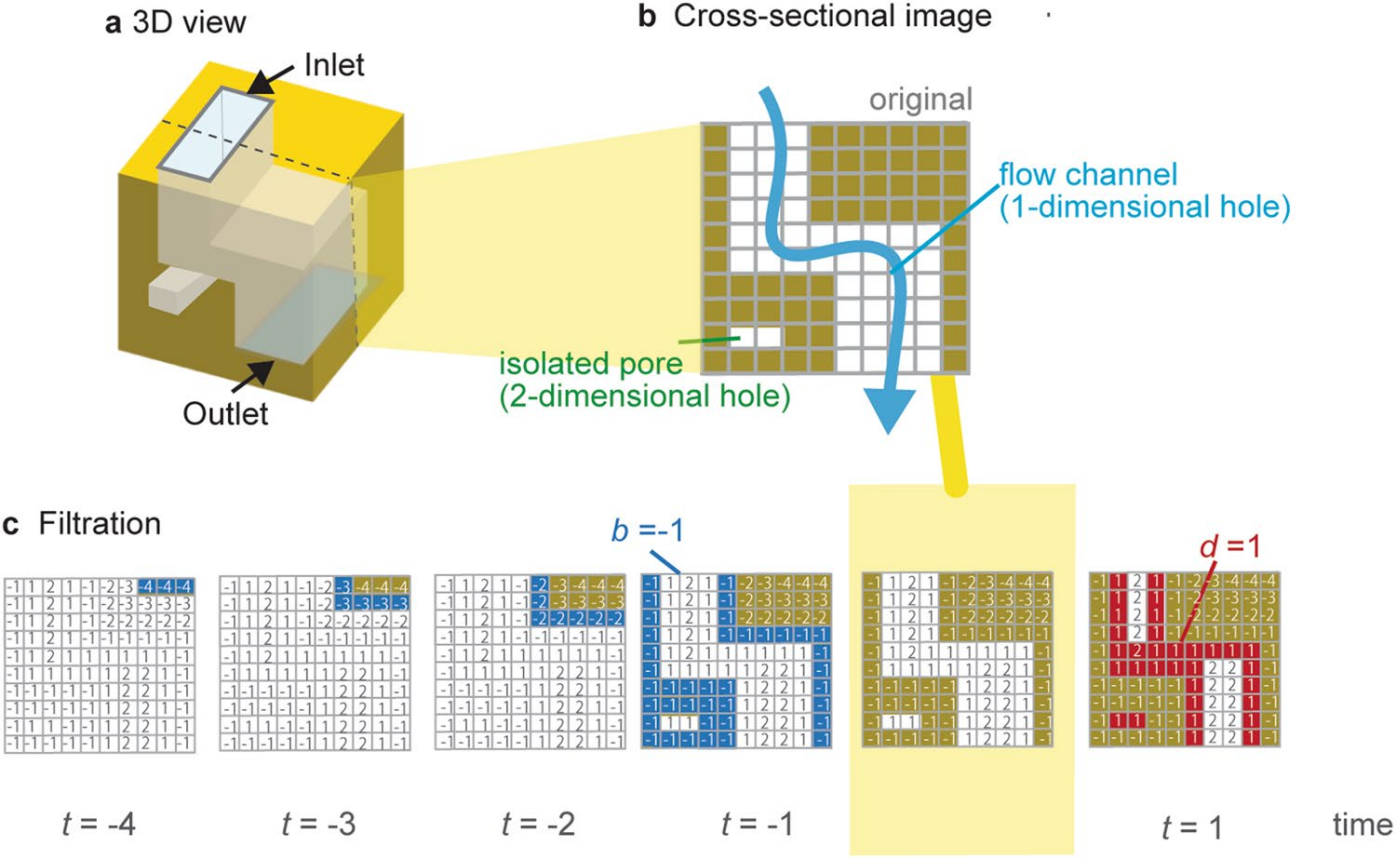
Persistent homology analysis for fracture networks by HomCloud^32^. (**a**) 3D view and (**b**) cross-sectional image of fracture network with a flow channel (light blue arrow) and an isolated pore (shown as green). (**c**) Schematic of filtration process. White grids express void spaces. Blue grids are the grids that were removed during the current thinning iteration. Red grids are the grids that were added during the thickening process.

An example of a fractured rock model with a flow channel connecting an inlet to an outlet is shown in Fig. 1a. The yellow area is a solid skeleton, while the white area is fractures forming void spaces. The connecting fractures from the top (inlet) to the bottom (outlet) can be a flow channel. In persistent homology analysis, such a structure is recognized as “hole” and quantified as a 1-dimensional hole. Additionally, a discrete island (i.e., connected component) is quantified as a 0-dimensional hole, and a ball (i.e., enclosed solid voids) is quantified as a 2-dimensional hole. The numbers of *k*-dimensional holes (the dimension of the *k*th homology vector space) are known to the *k*th Betti number (*b*_0_, *b*_1_, and *b*_2_). This study focuses on “hole” structures penetrating from an inlet to an outlet, which can be flow channels hence we only analyze 1-dimensional holes in this study.

One aspect of persistent homology analysis is that independent fractures are recognized as 2-dimensional holes, as shown in Fig. 1b. These independent fractures would not contribute to the fluid flow. Therefore, we can distinguish fractures that act as flow channels and independent fractures by 1-dimensional holes and 2-dimensional holes.

Much research has explored various applications of persistent homology in statistical data analysis of point cloud data^22,33,34^. Since the purpose of this study is to analyze the information of structures based on the image data (e.g., micro-CT images), binarized digital images were used for analysis. Jiang et al.^29^ applied persistent homology to analyzed rock pore geometries obtained from micro-CT images. The ﻿ rock pore geometries were first represented as sphere cloud data using a pore-network extraction method, then analyzed by ﻿calculating the Vietoris-Rips complex topology of the input sphere cloud data. We used an open software HomCloud (https://homcloud.dev/) to analyze binarized 3D images, which can obtain the information of persistent homology by calculating the Euclidian distance of 2D or 3D black and white images^32^.

Figure 1c shows an example of data process in our persistent homology analysis, called filtration^17^. In filtration, the solid skeletons (yellow parts) are made thinner or thicker, voxel-by-voxel. In HomCloud, the white pore areas were labelled with positive values, while the yellow solid areas were negative, hence the filtration labelling is symmetrical around the pore/solid interface. The process of thinning yellow voxels adjacent to white voxels is regarded as − 1, while the process of thickening yellow voxels adjacent to white voxels is regarded as + 1. When we reduce the time, the space eventually becomes empty. The nested sequence of the topological spaces from the empty space to the filled space is recorded. The times when the hole appears or disappears are called “birth time” or “death time”, expressed as “*b*” or “*d”*, respectively.

In filtration (Fig. 1c), a part of the flow channel is closed at *t* = 1. This closed point is the narrowest aperture in the flow channel. Taking advantage of this, the length of narrowest aperture can be obtained as death time *d* multiplied by two and its resolution *d*. (narrowest aperture = 2 × *d* × *d* = 2 × 1 × 5 = 10). Suzuki et al.^31^ used the values of death to detect the narrowest apertures of flow paths in 2D images and classified a large number of fracture structures. It has been known that the narrowest width in flow channels, which is called critical pore radius, correlates with permeability better than other pore radii^35–37^. Detecting narrowest aperture by persistent homology can therefore be useful to estimate the permeability.

The set of pairs (*b*_*i*_, *d*_*i*_) for *k*-dimensional holes is called *k*th persistence diagram, PD_*k*_. If pairs of negative *b* and positive *d* (*b* < 0 < *d*) are detected in PD_1_, the pairs suggest “hole” structures (i.e., flow channel) presenting in the original image. If there are multiple hole structures, multiple birth–death pairs are obtained in the *b* < 0 < *d* domain of PD_1_. Each value of *d* indicates each narrowest aperture of multiple fracture channels. The ability to link between the numbers of flow channels and its narrowest apertures is one of the strengths of persistent homology analysis.

**Figure 2:**
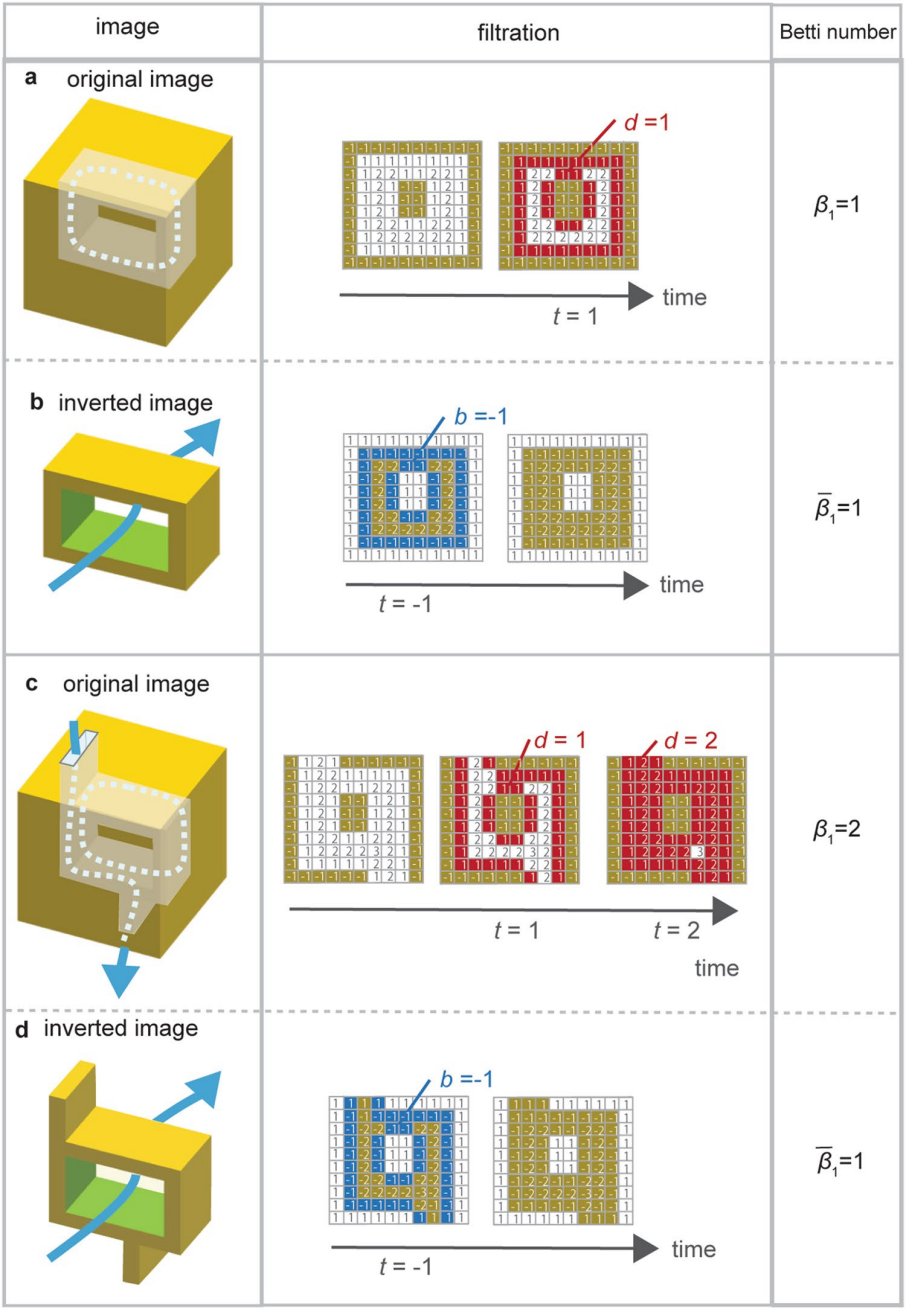
Detecting flow channels using inverted images (**a**) ring-shaped, internal void-structure that is not connected to the outside, and (**b**) its inverted image. (**c**) ring-shaped, internal void-structure with two channels that is connected to the outside and forms a flow channel. (**d**) Its inverted image. The left column shows 3D view of images. The center column describes processes of filtration. The right column lists Betti numbers *b*_1_*.*

Here is something to keep in mind. A 1-dimensional hole detected by persistent homology is a flow channel penetrating from an inlet to an outlet. At the same time, a ring-shaped, internal void-structure is also detected as a 1-dimensional hole. Figure 2a shows an example of a ring-shaped internal void structure. This structure does not connect to the outside (i.e., no flow channel). However, during filtration, the internal void space is closed at *t* = 1 (*d* = 1). If images include such hole structures, it would overestimate the number of flow channels. Now, let us prepare an inverted image that the yellow and white are reversed as shown in Fig. 2b. In filtration, a ring appears at *t* − 1, and the ring width is detected by the value of *b* (*b* = − 1). Therefore, we can expect to detect only the hole fractures that act as flow channels by subtracting the holes recognized in the inverted image from the holes recognized in the original image. We set the number of 1-dimensional holes (i.e., Betti number) obtained from the original and inverted figures to *b*_1_ and $\overline{\beta }_{1}$, respectively. The number of flow channels can be derived as $\beta_{1} - \overline{{\beta_{1} }} = 1 - 1 = 0$ as shown in Fig. 2a and 2b.

Another example is shown in Fig. 2c. This is a ring-shaped internal void structure with two channels that are connected to the outside. In this case, there are two 1-dimensional holes (*b*_1_ = 2) with *d* = 1 and *d* = 2. Figure 2d is the inverted image of Fig. 2c. There are a 1-dimensional holes (*b*_1_ = 1) with *d* = 1. By subtracting the holes recognized in the inverted image from the holes recognized in the original image, the number of flow channels can be calculated as $\beta_{1} - \overline{{\beta_{1} }} = 2 - 1 = 1$. At the same time, persistent homology analysis provides the narrowest aperture of the flow channel by *d* = 2. From these analyses, we estimate the number of channels and their narrowest aperture by using the inverted image in this study.

### Synthetic fracture network

Synthetic fracture networks were generated by using OpenSCAD (https://www.openscad.org/). We distributed multiple penny-shaped fractures by controlling the apertures, radii, numbers, and orientations of fractures to generate a fracture network^38^. By hollowing out the generated fracture network from a rectangular block, a fractured model where the void spaces were composed of the fracture network was created. This study characterizes one-dimensional flow. The top surface was an inlet, and the bottom surface was an outlet. The fractures were connected from the top to the bottom surfaces. The side boundaries were closed and impermeable.

**Figure 3:**
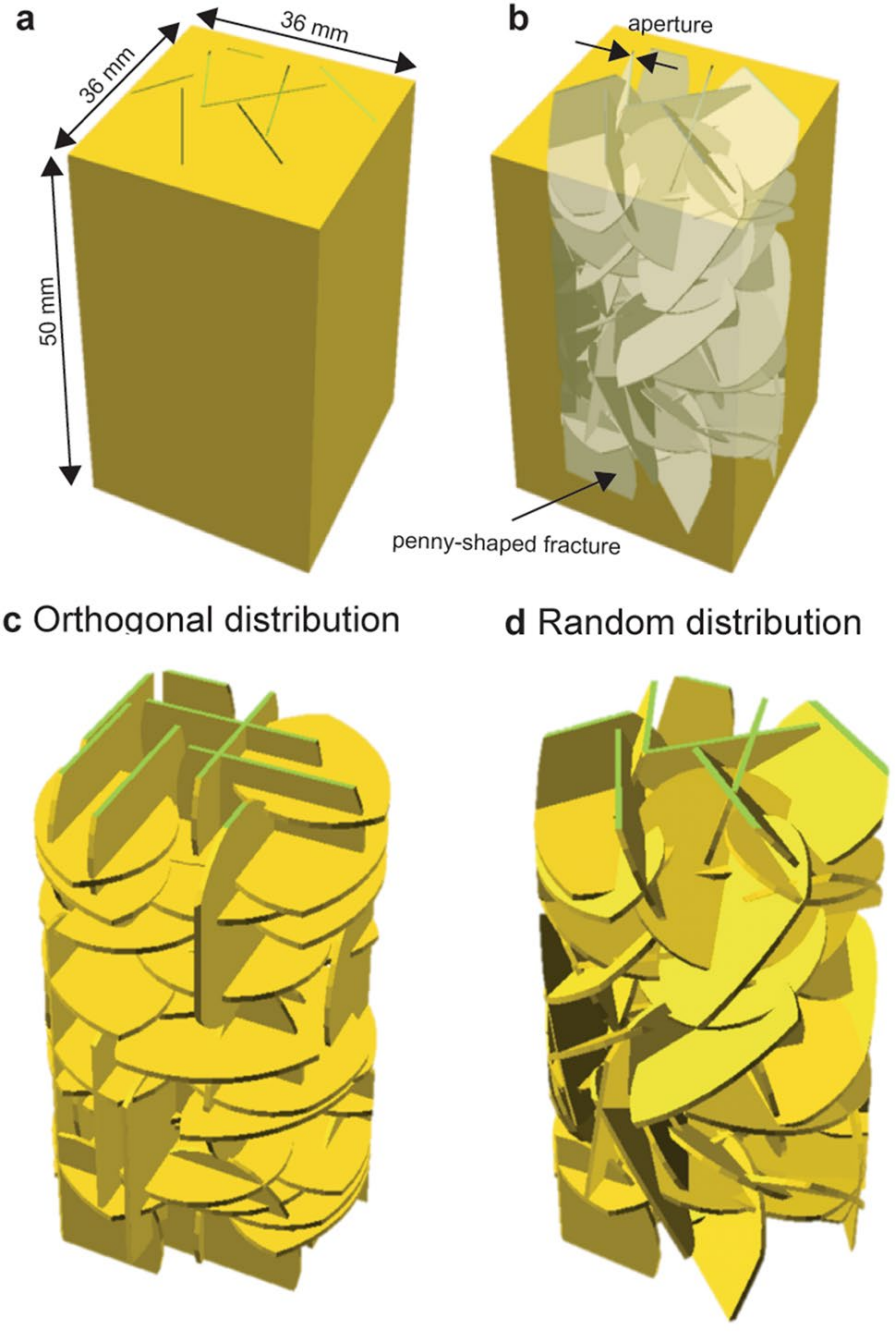
Fractured models. (**a**) Outside and (**b**) inside of model. (**c**) Orthogonal distribution and (**d**) random distribution of fracture networks.

The fractured model is shown in Fig. 3. Figure 3a and b are the outside and the inside of the model. The fracture orientation was either orthogonal or random. The orthogonal models distributed perpendicular or horizontal fractures to the flow direction (Fig. 3c), while the random models distributed fractures by random numbers (Fig. 3d). We prepared nine orthogonal models and seven random models. The model parameters for each model are listed in Table 1.

**Table 1:** Fracture network parameters and results of permeability.

Model	Fracture network parameters				Simulation result	Estimation from PH analysis
	Diameter (mm)	Aperture (mm)	Fracture density parameter	Number of fractures		
**Orthogonal**						
O1	10	0.2	3000	77	1.04 × 10^−10^	2.47 × 10^−10^
O2	10	0.6	3000	77	3.03 × 10^−9^	3.43 × 10^−9^
O3	10	1.0	3000	77	1.31 × 10^−8^	1.55 × 10^−8^
O4	5	0.2–1	6100	234	1.58 × 10^−10^	2.68 × 10^−10^
O5	10	0.2–1	1720	66	4.00 × 10^−10^	7.18 × 10^−10^
O6	5	0.2–1	2020	251	3.50 × 10^−10^	2.70 × 10^−10^
O7	5	0.2–1	13,000	230	1.12 × 10^−10^	2.16 × 10^−10^
O8	5–25	0.2–1	895	73	3.15 × 10^−9^	2.20 × 10^−9^
**Random**						
R1	10	0.2	2000	77	1.01 × 10^−10^	2.36 × 10^−10^
R2	10	0.6	2000	77	2.90 × 10^−9^	3.46 × 10^−9^
R3	10	1.0	2000	77	1.28 × 10^−8^	1.39 × 10^−8^
R4	10	0.2	1000	38	3.42 × 10^−11^	1.12 × 10^−10^
R5	10	0.2	3000	115	1.75 × 10^−10^	3.70 × 10^−10^
R6	25	1.0	280	11	5.21 × 10^−9^	4.03 × 10^−9^
R7	5	0.2	11,950	459	1.30 × 10^−10^	1.36 × 10^−10^

The correlation coefficients between simulation results and PH estimation for orthogonal distribution and random distribution were 0.994 and 0.992, respectively.

### Estimation of fracture numbers and apertures by persistent homology

3D image data of each fractured model (36 mm × 36 mm × 50 mm with a voxel resolution *d* of 0.1 mm) were binarized and analyzed by persistent homology using HomCloud^32^. The image size was 360 × 360 × 500 voxels.

**Figure 4:**
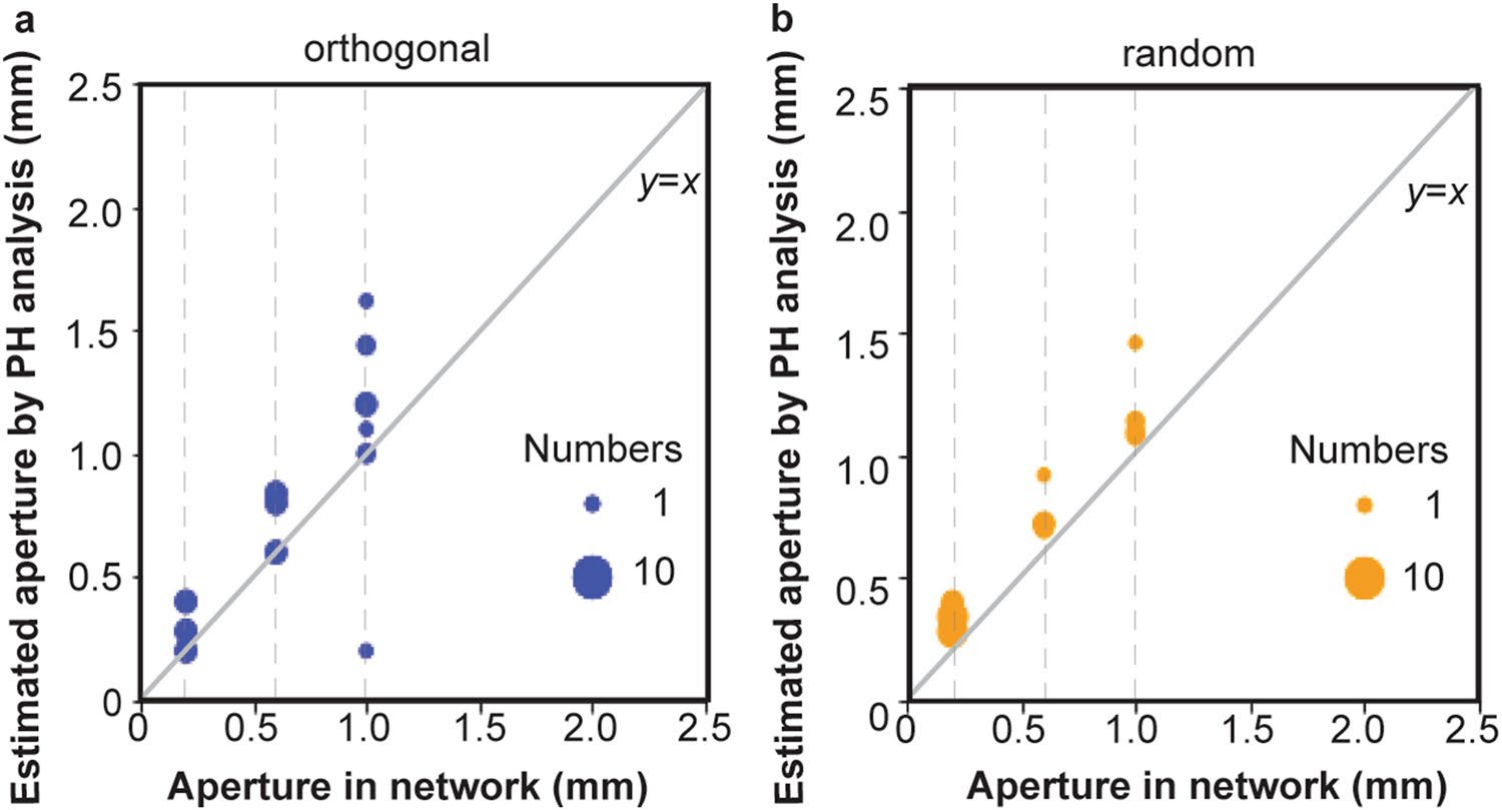
Estimation of fracture apertures by persistent homology (PH) analysis. (**a**) Orthogonal fracture networks and (**b**) random fracture networks.

The estimated narrowest fracture apertures based on the persistent homology analysis are shown in Fig. 4. Fracture networks (O1–O3, R1–R3) distributes a single value of fracture aperture of 0.2 mm, 0.6 mm, and 1.0 mm, respectively. Figure 4a and b shows the results for the orthogonal and the random fracture networks, respectively. As mentioned before, the narrowest apertures in each flow channel were calculated as 2*d*_*i*_*d* in persistent homology analysis. The values given in each network (0.2 mm, 0.6 mm, 1.0 mm) are compared with the estimated narrowest apertures (2*d*_*i*_*d*). The sizes of the circles represent the number of birth–death pairs with *d*_*i*_. As shown in Fig. 4, the estimated narrowest apertures are equal or relatively larger than the actual values given in the network. Most of results are between one or two times larger than the actual values. Since persistent homology estimates the aperture of flow paths, where there were multiple fractures overlapping each other, the aperture of the flow path would be thicker than the aperture of the fractures at these points. For this reason, the estimated values can be larger than the actual values given to the fracture network. On the other hand, since the image of the fracture network was converted to voxel data, the surface of the fracture distributed diagonally to the voxel may be rugged and larger than the actual fracture. This error associated with image processing can be resolved by increasing the resolution.

In the orthogonal network model as shown in Fig. 4a, there is a point in the 1.0 mm network where the fracture aperture was calculated as 0.2 mm. This would be a result of representing the fracture surfaces as an image, i.e. a discretized array of voxels. The situation with such errors is expected to occur when other images are analyzed, but errors with small values of apertures do not have a large impact on the analysis, as will be shown later.

### Derivation of permeability

We use Eq. (2) to derive permeability, which can be originally calculated by comparing the Stokes equation with the Darcy’s law. If Assuming that a fracture is a smooth and parallel plate with the aperture of *h* and that there is a uniform pressure gradient in one direction within the plane of the fracture, the total volumetric flowrate in the fracture can be written as

3 $$ Q_{x} = - \frac{{wh^{3} }}{12\mu }\frac{dP}{{dx}} $$

where *w* is the width of the fracture, perpendicular to the flow direction. *h* is the aperture, and *μ* is the water viscosity, *dP/dx* is the pressure gradient. Darcy's law describes one‐dimensional fluid flow through porous media as

4 $$ Q_{x} = - \frac{KA}{\mu }\frac{dP}{{dx}} $$

where *A* is the cross-sectional area. Comparison of Eqs. (3) and (4) shows that the permeability of the fracture can be identified as

5 $$ K = - \frac{{wh^{3} }}{12A} $$

If the cross-sectional areas of the inlet and outlet are assumed to be *wh*, Eq. (5) becomes Eq. (2). If we consider the case of parallel multiple channels, the permeability can be derived in the following equation

6 $$ K = \mathop \sum \limits_{i = 1}^{N} \frac{{w_{i} h_{i}^{3} }}{12A} $$

where *A* is the surface area of the cross section of the medium, and *N* is the number of flow channels. *w*_*i*_ is the depth of flow channel, and *h*_*i*_ is the aperture of the flow channel *i, i* = 1, …, *N*. There is an unknown parameter *w*_*i*_ in Eq. (6). The 3D voxel data can be regarded as a series of 2D cross-sectional images. The 2D cross-sectional image data provides total area of pore space, *A*_*p*_ in each layer. If we introduce effective depth $\overline{w}$ that is the same for all flow channels, $\overline{w}$ can be derived by $\overline{w} = \frac{{{\text{min}}\left( {A_{p} } \right)}}{{\mathop \sum \nolimits_{i = 1}^{N} h_{i} }}$ where ${\text{min}}\left( {A_{p} } \right)$ is the minimum of total area of pore space for all layers. The number of flow channels *N* was estimated from the number of birth–death pairs, and the aperture *h*_*i*_ was estimated as 2*d*_*i*_*d* in persistent homology analysis. Thus, Eq. (6) can be written as follows

7 $$ K = \frac{{\overline{w}}}{12A}\mathop \sum \limits_{i = 1}^{N} (2d_{i} \delta )^{3} $$

As mentioned earlier, it is expected that small apertures of flow paths may be detected due to errors associated with the image processing. However, as shown in Eq. (7), the permeability is determined by the sum of the cube of the apertures, so the apertures of larger fractures will affect the calculation of the permeability. Therefore, it can be said that the error associated with the image processing is negligible.

### Estimation of permeability from persistent homology analysis

**Figure 5:**
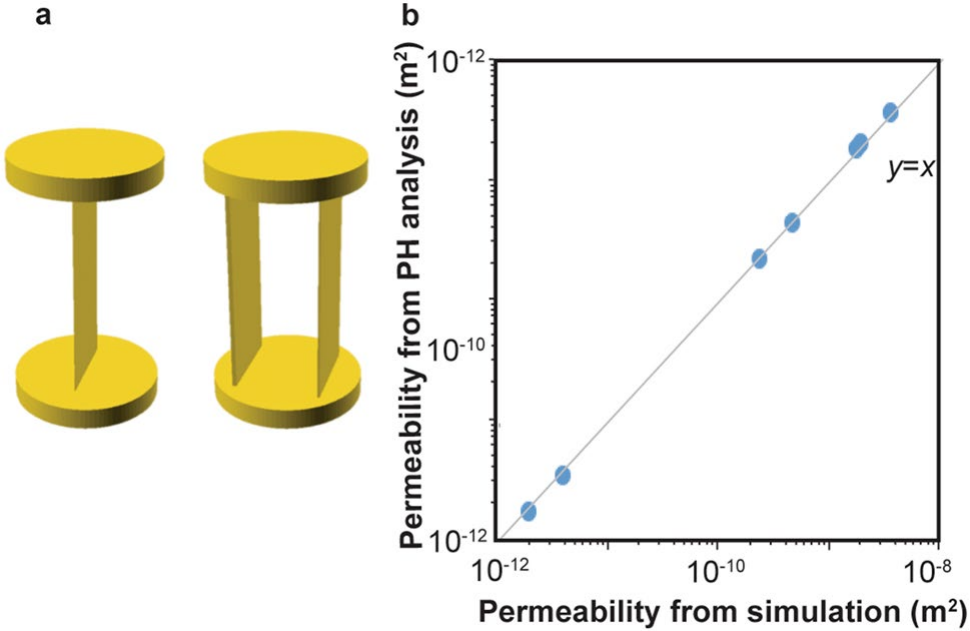
Validation with simple models. (**a**) One or two fracture penetrating the model. The top and bottom are the inlet and the outlet. (**b**) Comparison of permeability between persistent homology (PH) analysis and direct simulation. The calculated permeability is listed in Table 2.

Before applying complex fracture networks, we validated Eq. (7) and our simulation with simple fracture models. Simple models with one or two fractures penetrating from an inlet to an outlet were used (see Fig. 5a). Apertures and number of fractures in each model are listed in Table 2. Direct flow simulation with the same fracture network was conducted in OpenFOAM (https://www.openfoam.com/). We could obtain volumetric flow rate and pressure gradient between the inlet and the outlet to calculate equivalent permeability based on Darcy’s law. Comparison of permeability between flow simulation and persistent homology analysis is shown in Fig. 5b, and listed in Table 2. To quantitatively validate our estimation, a Pearson correlation coefficient between the simulation results and estimation from persistent homology analysis was calculated. When the correlation coefficient is close to 1, we can say that two set of data has high positive correlation. The correlation coefficient between simulation results and PH estimation for the simple parallel model was 0.9996. The persistent homology estimation is in very good agreement with the simulation results. The results show that for such a simple system, persistent homology can estimate the permeability well using Eq. (7).

**Table 2:** Estimation for simple parallel model. The correlation coefficient between simulation results and PH estimation was 0.9996.

Model	Aperture (mm)	Number of fractures	d	$\beta_{1} - \overline{{\beta_{1} }}$	Simulation result	Estimation from PH analysis
S1	1.0	1	10	1	1.80 × 10^−9^	1.80 × 10^−9^
S2	1.0 and 1.0	2	10 and 10	2	3.66 × 10^−9^	3.47 × 10^−9^
S3	0.5	1	5	1	2.39 × 10^−10^	2.17 × 10^−10^
S4	0.5 and 0.5	2	5 and 5	2	4.79 × 10^−10^	4.33 × 10^−10^
S5	0.1	1	1	1	1.95 × 10^−12^	1.73 × 10^−12^
S6	0.1 and 0.1	2	1 and 1	2	3.89 × 10^−12^	3.47 × 10^−12^
S7	0.5 and 1.0	2	5 and 10	2	2.00 × 10^−9^	1.95 × 10^−9^

**Figure 6:**
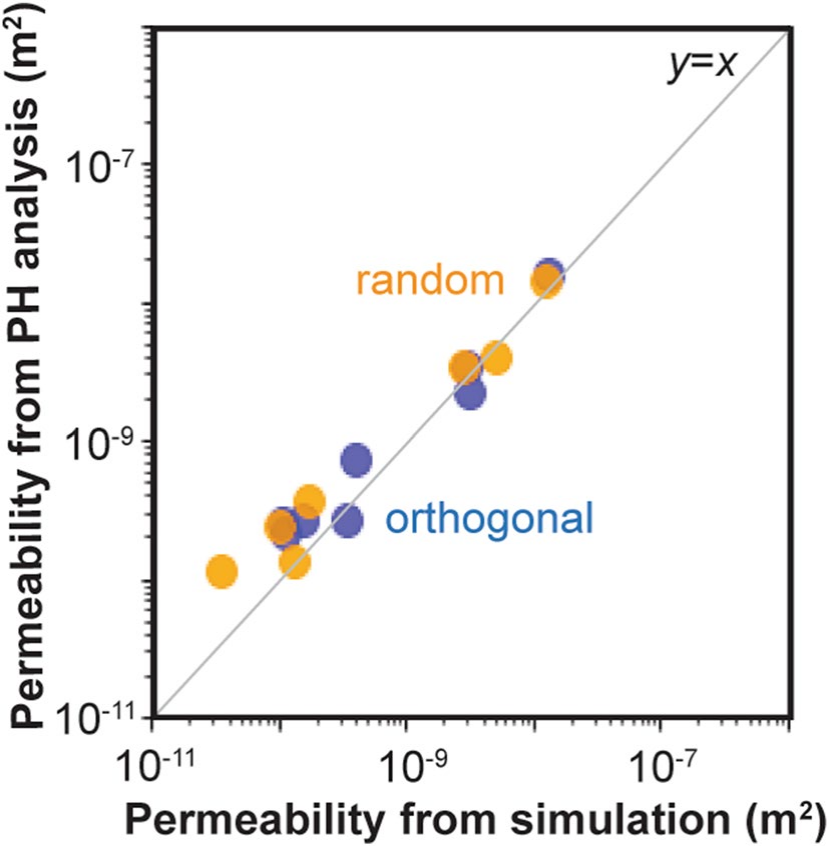
Estimation of permeability by persistent homology (PH) analysis for orthogonal fracture networks (blue) and random fracture networks (orange). The calculated permeability is listed in Table 1.

Next, we applied Eq. (7) to complex fracture networks listed in Table 1. Comparison of permeability between flow simulation and persistent homology analysis is shown in Fig. 6. The correlation coefficients between the simulation results and the estimation from persistent homology for orthogonal distribution and random distribution were 0.994 and 0.992, respectively. The estimated values were highly correlated to the simulation values. The estimation is in reasonable agreement with the simulation results.

**Figure 7:**
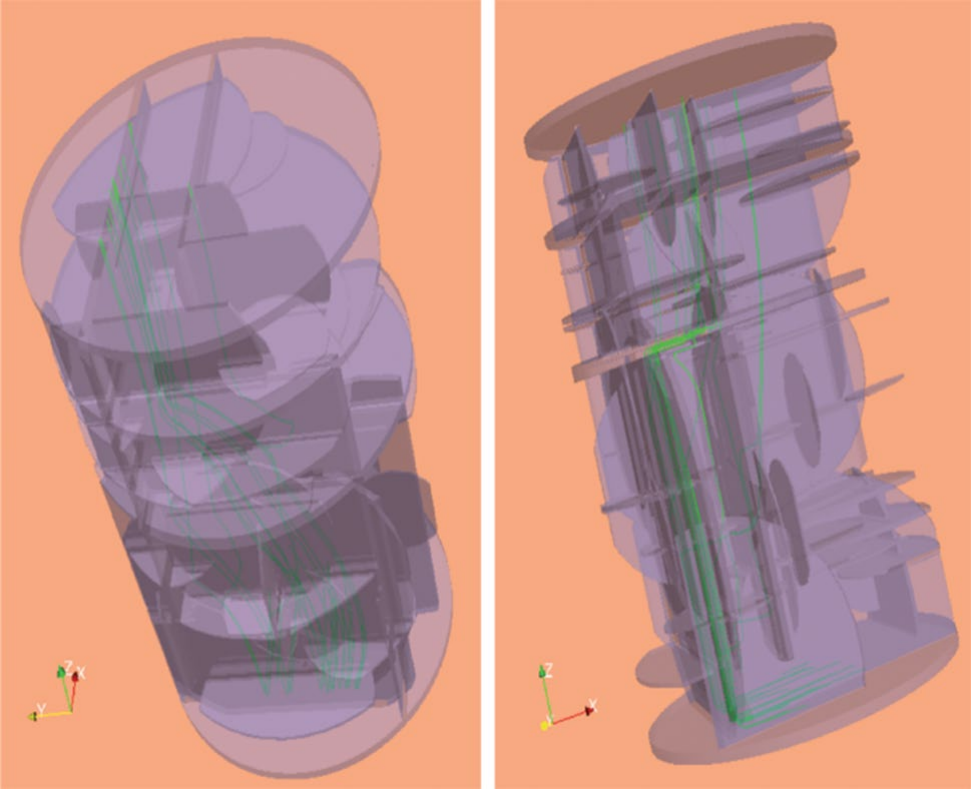
Streamlines (green lines) in fracture network simulated in OpenFOAM.

There is a limitation of Eq. (7). Equation (7) is based on a parallel plate model, so the flow is assumed to be straight. If there is tortuosity in a flow channel, the flow length will be longer, and the estimated permeability may be larger than the true value. Figure 7 shows streamlines in model O8 colored as green. We can see that the streamlines are winding and flowing. Keep in mind the fact that tortuosity was not taken into account in Eq. (7).

**Figure 8:**
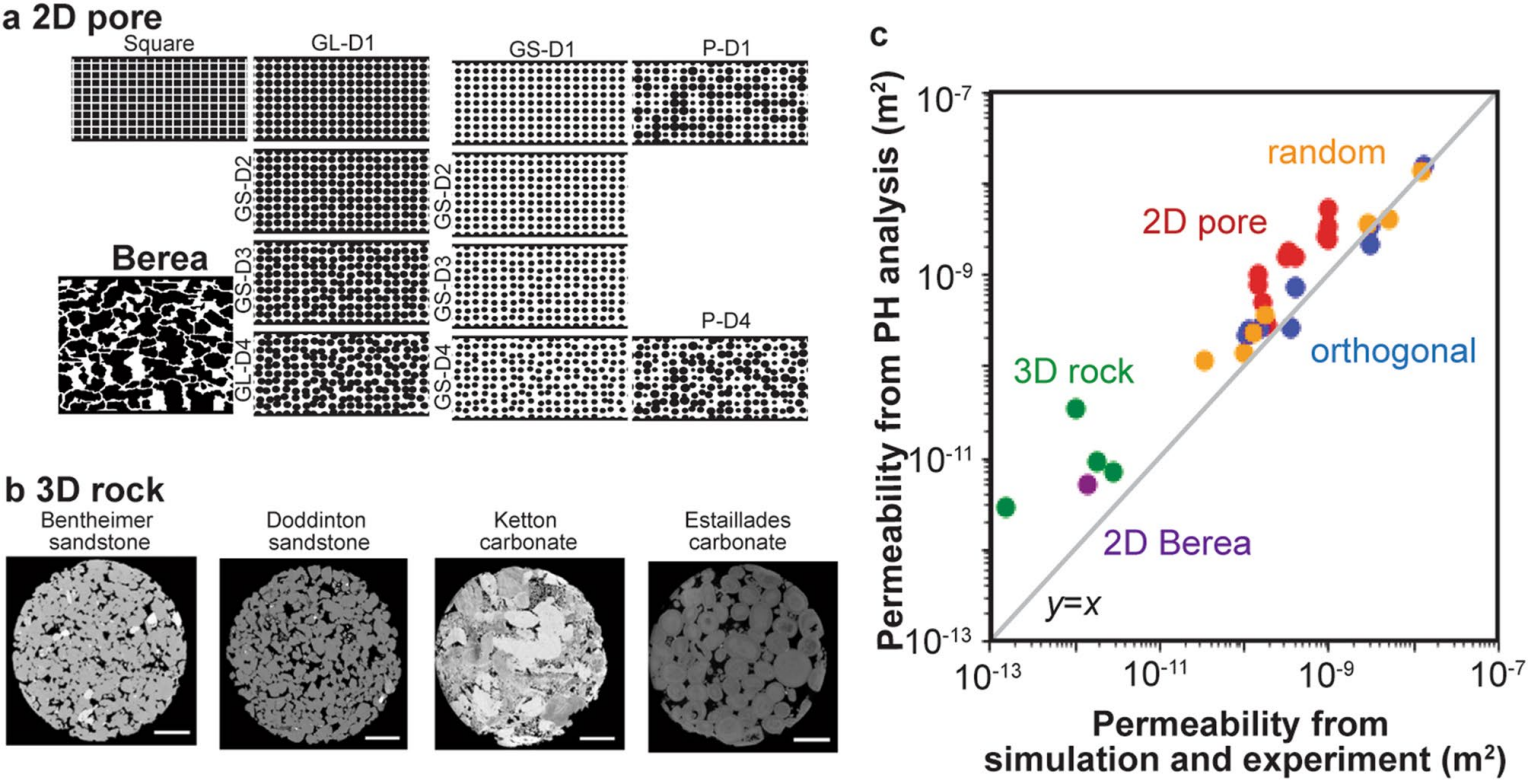
Estimation of permeability by persistent homology (PH) analysis. (**a**) 2D images from ﻿Mehmani and Hamdi^39^, (**b**) 3D rock images from Andrew et al.^40^, and (**c**) comparison with direct simulation and experiment. The values are listed in Table 3.

We also applied persistent homology analysis to other cases. Mehmani and Mamdi^39^ conducted high-fidelity direct numerical simulation of the two-dimensional micromodel to develop their pore network models. We used their 2D image data as shown in Fig. 8a and their results from direct numerical simulation. Comparison with persistent homology analysis is plotted with red dots for regular pore structures and with purple dots for Berea sandstone in Fig. 8c. The correlation coefficients between the simulation results and the estimation from persistent homology was 0.887. There is a fair correlation between the simulation results and the estimated values. These results suggest that the proposed analysis can be used for two-dimensional flow.

**Table 3:** Information of 2D pore^39^ and 3D rock images^40^ and their permeability.

Model	Image size (pixels)	Domain size (mm)	Simulation result	Estimation from PH analysis
**2D pore**				
Square	3000 × 1500	﻿20 mm × 10 mm × 0.2 mm	3.13 × 10^−10^	1.54 × 10^−9^
GL-D1			1.92 × 10^−10^	2.74 × 10^−10^
GL-D2			1.70 × 10^−10^	4.96 × 10^−10^
GL-D3			1.47 × 10^−10^	7.98 × 10^−10^
GL-D4			1.44 × 10^−10^	9.71 × 10^−10^
GS-D1			9.77 × 10^−10^	5.19 × 10^−9^
GS-D2			9.75 × 10^−10^	3.34 × 10^−9^
GS-D3			9.60 × 10^−10^	2.43 × 10^−9^
GS-D4			9.18 × 10^−10^	2.67 × 10^−9^
P-D1			3.25 × 10^–10^	1.75 × 10^−9^
P-D4			4.01 × 10^−10^	1.53 × 10^−9^
Berea	2900 × 2320	1.774 mm × 1.418 mm × 0.02454 mm	1.45 × 10^−12^	5.06 × 10^−12^

Model	Image size (voxels)	Resolution (um/px)	Experimental result	Estimation from PH analysis
**3D rock**				
Doddington	300 × 300 × 300	2.6929	1.04 × 10^−12^	3.37 × 10^−11^
Bentheimer		3.0035	1.88 × 10^−12^	9.02 × 10^−11^
Ketton		3.00006	2.81 × 10^−12^	6.86 × 10^−11^
Estaillades		﻿3.31136	1.49 × 10^−13^	2.90 × 10^−11^

The correlation coefficients between the simulation results and estimations from persistent homology analysis for 2D pore models and 3D rock models were 0.887 and 0.764, respectively.

Andrew et al.^40^ used X-ray microtomography to obtain four types of 3D rock image data, and they conducted flow experiments to measure the permeability. The X-ray microtomography images of the rocks are shown in Fig. 8b. Comparison with the experimental results is plotted as green dots in Fig. 8c. The correlation coefficients between the simulation results and the estimation from persistent homology was 0.764. The estimation has a slight correlation with the simulation results, and the estimated values are larger than the experimental results. As mentioned before, Eq. (7) does not consider the effect of tortuosity. Muljadi et al.^41^ calculated the tortuosity from the same Bentheimer sandstone and the Estaillades carbonate images as 1.52 and 1.91, respectively. If we take the tortuosity into account, the estimates of the permeability will be close to the experimental values. The calculation of tortuosity in Muljadi et al.^41^ used the flow velocity^42,43^. In contrast, the goal of this study is to estimate flow properties without flow simulation, so that we need to obtain tortuosity in a different way based on image analysis. Correlation between tortuosity and persistent homology parameters would be explored in future studies.

## Discussions

Persistent homology analysis of fracture networks could estimate opening aperture distributions of flow channels and estimated permeability with the same order of magnitude as the permeability derived from the flow simulation. Using this method, flow characteristics can be estimated from the image data without the need for fluid flow simulation. This could make the analysis of fracture networks quicker. In this study, the longest direct flow simulation took 72 h to generate a sufficiently high-resolution computational mesh then solve the Navier–Stokes equations, using 320 processors with a maximum of 282 GB of memory in a supercomputational system. In contrast, persistent homology was able to calculate the model in less than 10 min with 16 GB of memory using a desktop workstation AMD Ryzen 9 5950X.

Several approaches^44–46^ based on discrete fracture network represent fractures as ellipses or rectangles in networks based on Eq. (2). Focusing on the fractures themselves is suitable for fractured rock bodies, but it may be difficult to optimize the model because of the increase in number of parameters when the fractures are finer or when the body is regarded as a porous medium. In this study, we focus on the flow channels by persistent homology instead of individual fractures. Therefore, we can apply the method regardless of porous or fractured rocks.

Recently, some studies have been published to investigate the relationship between porous structures and flow by persistent homology^29,30,47,48^. Most of them were machine learning approaches that put a large number of parameters into a black box. In contrast, since our approach focuses on flow-channel structures, permeability can be calculated by the simple and easy principle. We used the synthetic fracture networks as well as natural rocks. Although the estimation errors were relatively large for 3D rocks, it was shown that a simple model such as Eq. (7) can provide reasonably close estimates.

Ushizima et al.^26^ estimates permeability of porous rocks by using Reeb graphs to ﻿represent the pore networks. They use persistent homology to distinguish between significant and noisy pore spaces, and to supplement the Reeb graphs. In fact, the Reeb graph and persistent homology were used independently and separately. We think that using Reeb graphs is a good direction to go to the next step.

We have succeeded in modeling physical phenomena from image data based on the topological data analysis. The method could be applied also to a wide range of porous media including artificial devices. It is expected to be applicable not only to estimate flow properties, but also to characterize different transport phenomena, such as mass transfer, electrical and magnetic flows.

## Method

### Persistent homology analysis

STL files of synthetic fracture networks were generated by using OpenSCAD, and the STL files were converted to the cross-sectional images in 36 mm × 36 mm × 50 mm with a voxel resolution of 0.1 mm in Autodesk Netfabb. To eliminate some unexpected noises, all the images were blurred in XnConvert. The png files of the image data were analyzed in HomCloud^63^. When there were small differences between birth and death of PH_1_, the hole structure may appear during the image analysis. Thus, we neglected the result with *d*_*i*_ − *b*_*i*_ < 2 were eliminated.

### Direct flow simulation

**Figure 9:**
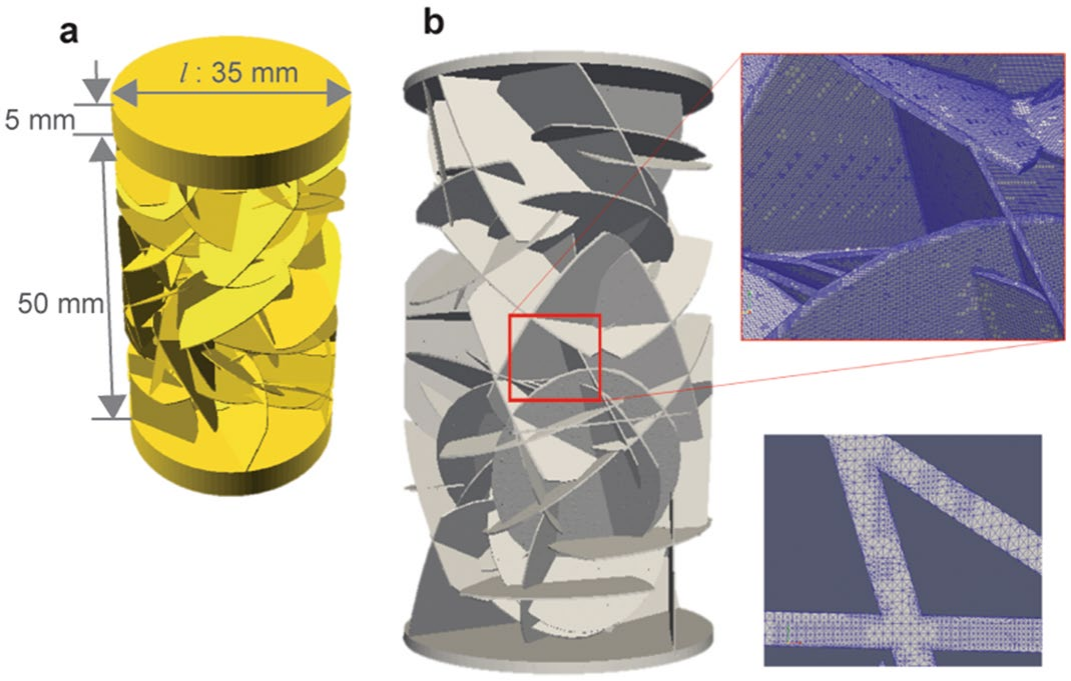
Simulation in OpenFOAM. (**a**) Fracture network with upper and lower boundaries. (**b**) Discretized model.

Flow behaviors in the rock models were simulated in OpenFOAM (ver. 4.1)^34^, which performs flow calculations based on the Navier–Stokes equation. The simulation used an STL file of the network model, in which the upper and lower boundaries were added, as shown in Fig. 9a. Previous study validated the flow model with fracture networks similar to the rock model in this study by creating test specimens from a 3D printer and conducting flow experiment^49,50^.

First, the meshes of the region were prepared (Fig. 9b), and flow calculation of the loaded STL file were performed using a steady-state turbulence solver for incompressible fluids SIMPLE (Semi-Implicit Method for Pressure Linked Equations) method. In this study, we set the flow rate to 1.75 × 10^−7^ m^3^/s, fluid viscosity to 9.32 × 10^−4^ Pa s, and fluid density to 997.5 kg/m^3^.

## Data Availability

The data that support the findings of this study are available in https://doi.org/10.6084/m9.figshare.14110262, https://doi.org/10.6084/m9.figshare.14110208, https://doi.org/10.6084/m9.figshare.14113439.
